# Strategies to promote the completion of patient-reported outcome measures by culturally and linguistically diverse and Indigenous Peoples in clinical care settings: A systematic review

**DOI:** 10.1007/s11136-025-03913-2

**Published:** 2025-02-08

**Authors:** Jessica Nikolovski, Bora Kim, Rachael L. Morton, Rebecca Mercieca-Bebber, Jean-Frédéric Levesque, Melissa Tinsley, Kim Sutherland, Brad Rossiter, Margaret Fagan, Gill Hartas, Claudia Rutherford

**Affiliations:** 1 NHMRC Clinical Trials Centre, University of Sydney, Camperdown, Australia; 2 Sydney Quality of Life Office (SQOLO), Susan Wakil School of Nursing and Midwifery, Faculty of Medicine and Health, University of Sydney, Camperdown, Australia; 3 The Daffodil Centre, The University of Sydney, a Joint Venture with Cancer Council New South Wales, Sydney, NSW Australia; 4 Agency for Clinical Innovation, Sydney, Australia; 5 Office for Health and Medical Research, NSW Ministry of Health, Sydney, Australia

**Keywords:** Patient-reported outcome measures, Clinical care settings, Culturally and linguistically diverse, Indigenous Peoples, Systematic review

## Abstract

**Purpose:**

There is evidence of low completion of patient-reported outcome measures (PROMs) by people from culturally and linguistically diverse (CALD) backgrounds and Indigenous Peoples with chronic health conditions. We aimed to systematically identify ways to support and promote PROM completion by CALD communities and Indigenous Peoples in clinical care settings.

**Methods:**

We searched Medline, Embase, Scopus, Web of Science Core Collections and CINAHL databases from 1 January 2000 to 19 September 2024. Primary studies were included if they focused on ways to support and promote PROM completion in the care of CALD and Indigenous populations in clinical care settings. The quality of the included papers was appraised independently by two reviewers, using the Critical Appraisal Skills Programme (CASP) and Mixed Methods Appraisal Tool (MMAT). Data were analysed thematically. PROSPERO registration: CRD42023469317.

**Results:**

Of 13,450 title/abstracts retrieved, five papers met eligibility. Strategies to promote PROM completion by Indigenous Peoples included (1) providing training to patients about what PROMs are (2) offering verbal modes of completion and (3) community consultation during design, development, and implementation of PROMs to ensure culturally appropriate and sensitive PROMs are used. Strategies to promote completion by people who are CALD included (1) providing information about how to use electronic PROMs, (2) facilitating self-completion, (3) offering different modes of completion (paper-based, digital), (4) increasing availability of culturally and linguistically appropriate PROM translations, and (5) system-wide financial and administrative support to use translated PROMs.

**Conclusion:**

Few studies reported strategies to support the completion of PROMs by people from CALD backgrounds and/or Indigenous Peoples. Adequate training, planning (including community consultation), resourcing, and financial support are required to encourage people who are CALD and Indigenous Peoples to participate in PROM initiatives globally.

## Abstract (plain language summary)

People from culturally and linguistically diverse (CALD) backgrounds and Indigenous Peoples can struggle to complete health surveys about how they feel, function and live their lives with their health conditions. This study looks at ways we can support people to complete health surveys. We found that we can help Indigenous Peoples to complete health surveys by teaching them about what health surveys are used for, offering verbal completion, and involving the community in designing health surveys. For people from a CALD background, useful ways included providing information about electronic health surveys and how to complete them. Our findings highlight that patients and healthcare staff need more training to use health surveys, community involvement is important when creating health surveys, and more resources are needed to help people from CALD backgrounds and Indigenous Peoples to complete health surveys. This will help their voices to be included in healthcare decision-making.

## Introduction

Chronic conditions are long-lasting and persistent medical conditions that impact a person’s health-related quality of life (HRQL) [1] and are responsible for 74% of all deaths globally [2].

It is reported that people from culturally and linguistically diverse (CALD) backgrounds and Indigenous Peoples experience higher rates of chronic conditions and worse health outcomes over time compared with their English-speaking counterparts [3] including poorer survival outcomes [4], HRQL and psychological wellbeing [5], and increased risk of hospitalisation and emergency department visits [6]. People from CALD backgrounds and Indigenous Peoples are historically marginalised, underserved and under-represented in healthcare policy and decision-making [7–10].

### Defining ‘CALD’ and IndigenousPeoples

The Australian Bureau of Statistics, in 1999, introduced the term “Cultural and Linguistic Diversity” to draw attention to multicultural populations in Australia, with a range of linguistic and cultural characteristics. In this study, CALD refers to people born in non-English speaking countries, and/or who do not speak English at home and/or have limited English proficiency [11].

Indigenous Peoples are distinct social and cultural groups sharing collective ancestral ties to the lands where they live, occupy or have been displaced. Different terms have been used to refer to Indigenous Peoples such as Aboriginal and Torres Strait Islander peoples, indigenous Australians/peoples, Australia’s indigenous peoples, First Nations, and traditional owners. For consistency when reporting findings the term “Indigenous Peoples” will be used [11].

### Contributing factors of poor health status for CALD populations

People who are CALD may have poor access to high-quality medical advice due to socioeconomic factors such as living long distances from health services, limited access to culturally appropriate health services, and out-of-pocket costs that are unaffordable [12].

Poorer health outcomes within CALD populations may be exacerbated by linguistic and cultural incompatibility during clinical encounters [13, 14]. Even when people speak enough English to navigate daily life, it can still be difficult to describe health concerns like pain [12], and understand medical terminology [12, 15]. Healthcare professionals report it is harder to assess and manage symptoms reported by people from CALD backgrounds due to communication barriers [16] and overestimating English proficiency [17].

### Contributing factors of poor health status for Indigenous Peoples

A systematic review of disparities in healthcare services amongst Indigenous Peoples in North America, Australia and New Zealand highlighted factors such as rural location and socioeconomic status contribute to poorer accessibility of health services [18]. Cultural conflicts between Western medicine and native spirituality on Indigenous People’s view of health and wellbeing [18] can lead to mistrust and underutilisation of healthcare services, further exacerbating health disparities.

### Improving quality of life for CALD and Indigenous Peoples

Patient-reported outcome measures (PROMs) enable the collection of self-reported information from patients about their health condition without interpretation from anyone else [19] and assess outcomes such as disease symptoms, treatment side-effects, and HRQL [20, 21]. In clinical practice they can be used to facilitate communication between patients and their clinicians [22] and monitor responses to treatment over time [23].

In clinical care settings, there is low PROM completion rates by people who are CALD and Indigenous Peoples [24]. This means evidence informing clinical decision-making and medicines regulatory decisions often exclude the voices of people from CALD backgrounds and Indigenous Peoples, impacting the validity and generalisability of research findings as participant samples often fail to represent diverse populations [25].

### Study aim

By synthesising existing research, this systematic review aims to (1) identify ways to support and promote PROM completion by people from CALD backgrounds and Indigenous Peoples, (2) use findings to offer evidence-based recommendations for healthcare providers and policymakers, and (3) put findings in the context of an existing program of PROMs collection implemented in New South Wales (NSW), Australia.

## Methods

This systematic review was conducted and reported according to the Preferred Reporting Items for Systematic Reviews and Meta-Analyses (PRISMA) statement (see Supplementary File 1) [11] and was registered on PROSPERO: CRD42023469317.The SPIDER (Sample, Phenomenon of Interest, Design, Evaluation/Outcome, Research type) tool was adopted to define key elements of the review question and inform the search strategy [26].

### Electronic searches

JN developed a comprehensive search strategy with advice from the project team and iterative consultations with an academic liaison librarian at the University of Sydney. The search was conducted by JN in Medline, Embase, Scopus, Web of Science Core Collections and CINHAL electronic databases from 1 January 2000 (the year that PROMs were introduced in the United States as a reimbursement tool for healthcare organisations [27]) to 19 September 2024 (date data extraction and analysis was completed). Alerts were enabled to inform any new studies up until data extraction and analysis was finalised. The search strategy included population terms such as “culturally and linguistically diverse”, “Non-English Speaking Background”, “Indigenous Peoples”; intervention terms such as “patient-reported outcome”; outcome terms like “strategies”; and study design terms like “qualitative” and “mixed methods”. These concepts (population, intervention, outcome, and study design) were combined using the Boolean term “AND”. There were no language or country restrictions. See Supplementary File 2 for the full search strategy.

JN searched the reference lists and authors of included papers to ensure no potentially relevant papers were missed. No additional papers were added from these searches. One full-text paper in Spanish was translated into English by the University of Sydney Library.

### Study selection and eligibility criteria

Retrieved papers were assessed against the eligibility criteria outlined in Table 1.

**Table 1:** Inclusion and exclusion criteria used to screen eligibility of identified studies using SPIDER (Sample, Phenomenon of Interest, Design, Evaluation/Outcome, research type) tool

Aspect	Inclusion criteria	Exclusion Criteria
Sample	• Population are people from a CALD background, defined as people born in non-English speaking countries, and/or who do not speak English at home and/or have limited English proficiency (LEP) *or* • Population are Indigenous Peoples, defined as the first and original inhabitants of the country in which the study is set *and* • Adult CALD and/or Indigenous Peoples with a chronic condition, or healthcare staff treating patients with chronic conditions, defined as a condition that persists and/or requires ongoing surveillance/monitoring and/or treatment and/or worsens over time and/or patient experiences long or late-term effects of treatment *or* • Healthcare staff who use translated PROMs and/or use PROMs for people who are CALD and/or Indigenous Peoples	• Population: <18 years of age and/or not CALD or Indigenous and/or does not have chronic condition.
Phenomenon of interest	• PROMs completed in clinical care settings. PROMs defined as self-reported information from patients about their health condition without interpretation from anyone else [18] and assess outcomes such as disease symptoms, treatment side-effects, and health-related quality of life	• Focused only on PREMs, not PROMs.
Design	• Qualitative, quantitative, mixed-methods, cohort	• n/a
Outcome	• Attitudes, perspectives and experiences of using PROMs	• Focus on validation of PROMs or hypothetical scenarios generated (PROMs not implemented).
Research type	• Primary, peer-reviewed research	• Reviews, editorials.

Abbreviations: CALD: culturally and linguistically diverse; PROMs: Patient-reported outcome measures; PREMs: patient-reported experience measures

### Screening

**Fig. 1:**
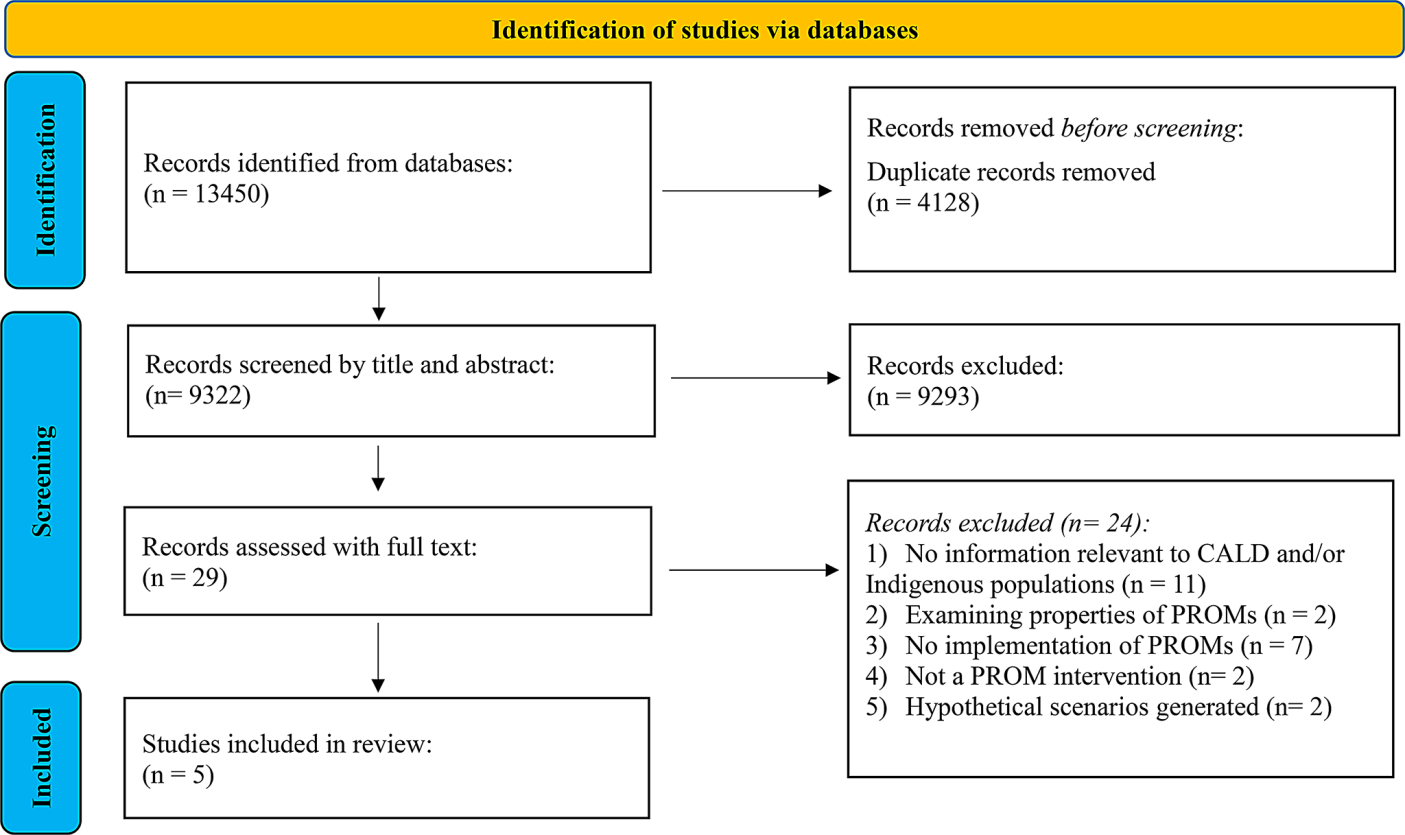
Preferred reporting items for systematic reviews and meta-analyses (PRISMA) flow-diagram of included papers

Search results from all databases were downloaded into Endnote 20 [28]. Following deduplication, remaining citations were exported into Covidence, a data management software used for systematic reviews (Veritas Health Innovation, Melbourne, Australia) [29]. One reviewer (JN) screened all retrieved titles and abstracts against the eligibility criteria. A second reviewer (BK) randomly screened 20% against the eligibility criteria to ensure inter-rater reliability. An a priori strategy was used where a threshold of 10% discrepancy was set; if discrepancies exceeded this level, an additional 10% of articles would be screened. The discrepancy rate between the first and second reviewers for the randomly selected 20% of articles was < 1%. Studies eligible for full-text review were independently assessed by JN and BK. Any disagreements about study eligibility were discussed between reviewers until a consensus was reached. Any disagreements were discussed with a third reviewer (CR). Study selection is summarised in the PRISMA flow chart (See Fig. 1).

### Data extraction and synthesis

Though some definitions of ‘CALD’ encompass Indigenous Peoples, we have synthesised and reported evidence for Indigenous Peoples separately as they are the first and original inhabitants, contributing uniquely to cultural and linguistic diversity in their respective countries.

#### Quantitative data

Data were extracted using a predetermined table in Microsoft Excel, including headings such as ‘information about PROM(s) used in the study’, ‘participants’, ‘study methods’, ‘data analysis’, ‘results’, ‘strategies for PROM completion’ and ‘perspectives and experiences of using PROMs’.

#### Qualitative data

Thematic analysis was used to synthesise qualitative findings [30]. Microsoft Word was used to group strategies to support and promote PROM completion from each paper, including representative quotes. Via an iterative process, JN and CR developed themes, and all authors were consulted to capture a range of clinical and health-service perspectives about the data.

### Assessment of methodological quality

#### Qualitative studies

To assess the methodological quality of qualitative studies, the Critical Appraisal Skills Programme (CASP) [31] assessment tool was chosen as it is a widely used tool for quality appraisal in health-related qualitative research, endorsed by the Cochrane Qualitative and Implementation Methods Group and recommended for novice qualitative researchers [32]. There are 10 questions across three sections with ‘yes’, ‘no’ and ‘cannot tell’ options: (A) Are the results of the study valid? (B) What are the results? (C) Will the results help locally?

#### Quantitative studies

To appraise the quality of mixed-method studies the Mixed Methods Appraisal Tool (MMAT) was used as it was developed for mixed-method systematic reviews [33]. There are five methodological quality criteria rated with ‘yes’, ‘no’ and ‘cannot tell’ options.

## Results

### Study inclusion

Three authors were contacted for further information to determine eligibility; these studies were excluded due to non-response (*n* = 2) or not meeting eligibility criteria (*n* = 1).

### Summary of included papers

Characteristics of the included papers are described in Table 2. Five studies were included which were conducted in the United States (*n* = 3) [34–36], Australia (*n* = 1) [37] and Europe (*n* = 1) [38] across a range of care settings, including academic, outpatient, inpatient and community centres. PROMs used in studies included the Patient-Reported Outcome Measurement Information System- 29 (PROMIS-29) [37], Problem Areas in Diabetes (PAID) [37], and MoPat2 [38] (specifically designed for the study). Three studies did not report which PROMs were used [34–36].

Study designs included qualitative (*n* = 4) [34–37] and mixed-methods(*n* = 1) [38]. CALD patient perspectives were reported in four studies [35–38] and perspectives of healthcare staff were reported in two studies [34, 38].

**Table 2:** Summary of included papers

First author (year of publication)	Location	Study design	Setting	Sample size	Patient or healthcare staff	Participant characteristics	PROM used
Allar (2022)	Boston, Massachusetts, USA.	Qualitative interviews	Academic medical centers	24	Healthcare staff	English speaking surgeons using translated PROMs	Not reported
Farina (2022)	Boston, Massachusetts, USA	Qualitative interviews	Cancer treatment and research facility	10	Patients	Spanish-speaking cancer patients	Not reported
Burgess (2022)	Shoalhaven, New South Wales, Australia	Qualitative interviews and focus groups	Community centres and participant homes	29	Patients	Aboriginal and Torres Strait Islander patients with diabetes	PROMIS-29, PAID
Long (2021)	Baltimore, Maryland, USA	Observation and qualitative interviews	Hand and upper extremity clinic	17	Patients	Non-English speaking patients attending clinic	Not reported
Soto-Rey (2018)	Europe	Quantitative survey	Outpatient care centre, dermatologic clinic, and university hospital	495 patients, 28 clinicians	Both	English, French, German, Italian, Polish, Spanish, and Turkish patients attending study clinics; clinicians employed at clinics	MoPat2

Abbreviations patient-reported outcome measures (PROMs); Patient-Reported Outcomes Measurement Information System- 29 (PROMIS-29); Problem Areas In Diabetes (PAID); United States of America (USA)

### Assessment of methodological quality

Given the scarcity of studies, we decided to include all studies in the review, despite the quality appraisal results, to ensure a comprehensive synthesis of the currently available evidence.

#### Qualitative papers

Table 3 shows the results from the quality appraisal of qualitative studies. Of note, 50% of the studies did not report sufficient information to determine if data analysis was rigorous (criteria 8), clearly describe if ethical issues were addressed (criteria 7) or describe if the relationship between researchers (criteria 6) was considered.

**Table 3:** Methodological quality of included qualitative papers using the critical appraisal skills programme (CASP) assessment tool

Question	Allar (2022)	Farina (2022)	Burgess (2022)	Long (2021)
1. Clear statement of aims	Yes	Yes	Yes	Yes
2. Appropriate qualitative methodology	Yes	Yes	Yes	Yes
3. Appropriate research design	Yes	Yes	Yes	Yes
4. Appropriate recruitment strategy	Yes	Yes	Yes	Yes
5. Data collection sufficiently addresses research issue	Yes	Yes	Yes	Yes
6. Relationship between researcher and participants considered	Yes	**No**	**No**	Yes
7. Ethical issues considered	**No**	**No**	Yes	Yes
8. Data analysis sufficiently rigorous	***Unclear***	***Unclear***	Yes	Yes
9. Clear statement of findings	Yes	Yes	Yes	Yes
10. How valuable is the research?	Valuable	Valuable	Valuable	Valuable

#### Quantitative and mixed-methods papers

The results from the quality appraisal of the mixed-method study [38] is shown in Table 4.

**Table 4:** Methodological quality of mixed-method papers using Mixed Methods Appraisal Tool (MMAT)

Study	Appraisal	Criteria					Criteria met	Final score
		1	2	3	4	5		
Soto-Rey (2018)	Mixed-method	No	Cannot tell	No	Cannot tell	No	0/5	0/5

Note. Quantitative criteria: (1) Is the sampling strategy relevant to address the research question? (2) Is the sample representative of the target population? (3) Are the measurements appropriate? (4) Is the risk of nonresponse bias low? (5) Is the statistical analysis appropriate to answer the research question? Qualitative Criteria: (1) Is the qualitative approach appropriate to answer the research question? (2) Are the qualitative data collection methods adequate to address the research question? (3) Are the findings adequately derived from the data? (4) Is the interpretation of results sufficiently substantiated by data? (5) Is there coherence between qualitative data sources, collection, analysis and interpretation? Mixed-methods criteria: (1) Is there an adequate rationale for using a mixed methods design to address the research question? (2) Are the different components of the study effectively integrated to answer the research question? (3) Are the outputs of the integration of qualitative and quantitative components adequately interpreted? (4) Are divergences and inconsistencies between quantitative and qualitative results adequately addressed? (5) Do the different components of the study adhere to the quality criteria of each tradition of the methods involved?

## Findings of the review

We identified five strategies to promote PROM completion in CALD and/or Indigenous populations, including one specific strategy reported by Indigenous Peoples, one specific strategy reported by CALD populations, one strategy reported by CALD and Indigenous Peoples, and two strategies from the perspective of healthcare staff about the use of translated PROMs in CALD populations. Notably, few specific strategies addressed how to promote PROM completion in these populations.

### Patient-level enablers and strategies

#### Theme: Patients had varied levels of digital and health literacy

Participants reported varied digital and health literacy which needed to be considered when developing strategies to promote PROM completion by people from CALD backgrounds and Indigenous Peoples.

#### Indigenous Peoples

An Aboriginal and Torres Strait Islander study participant expressed that PROMs administered verbally would facilitate completion by Aboriginal and Torres Strait Islander peoples because they *“come from the culture where we’re visual, we talk more (Aboriginal and Torres Strait Islander participant)”* [37].

Some Aboriginal and Torres Strait Islander participants reported PROMIS-29 was written at a level above average health literacy and included too much medical jargon, so terminology needed to be simplified and more straightforward to facilitate completion [37]:

*“I don’t know what diabetes is, you know, in plain English I don’t. All the doctor just give me a little tablet and I just took it, and I still don’t know what it means." (Aboriginal and Torres Strait Islander participant)* [37].

#### CALD populations

One qualitative study among 10 Spanish-speaking cancer patients reported interest in completing electronic PROMs (ePROMs) if there was a staff member available to help them understand how to use the ePROM platform, which contrasted English-speaking participants who largely reported disinterest in completing ePROMs [35].

ePROMs were accepted by non-English speaking dermatology patients in Europe if the platform was accessible in their home language [38]. For example, administrative and user functions were translated; patients could generate surveys in their preferred language (English, French, German, Italian, Polish, Spanish and Turkish) and respondent burden was reduced by limiting the number of questions patients needed to complete [38]. Older CALD participants were, on average, not willing to complete PROMs in the future and had difficulties using iPads compared with younger CALD participants [38]. In support of this, non-English speaking patients in the USA found ePROMs easier to complete if there were minimal system errors [36].

#### Theme: Willingness to complete PROMs depended on Indigenous People’s understanding of why they were asked to complete PROMs

Ensuring PROMs are culturally appropriate can help Indigenous Peoples understand why PROMs are used. As a strategy to increase PROMs uptake, Aboriginal and Torres Strait Islander participants with diabetes called for community representatives to be included in all stages of research development and conduct to ensure the chosen PROMs aligned with their cultural needs: *“the questions need to be looked out from us, like from a cultural way”(Aboriginal and Torres Strait Islander participant)* [37]. PROM completion would have been encouraged if there were specific questions about family, culture, and the impact that the Stolen Generations has had on Aboriginal health [37]. Aboriginal and Torres Strait Islander participants believed PROMIS-29 was written with the assumption they had easy access to services, which is not true for many of them [37]. This finding was not reported by CALD populations in the included studies.

#### Theme: CALD populations require support to understand and complete PROMs

In several studies, staff, carers, and family tended to help CALD patients complete PROMs by translating questions, recording responses, or providing information about what PRO scores meant [34–36]. However, when staff assisted people from CALD backgrounds to complete PROMs with a live phone interpreter, staff did not always accurately read out the PROM questions and responses [36].

### Healthcare staff enablers and strategies

Notably, no included studies reported the perspectives of healthcare staff that used PROMs with Indigenous Peoples. Both studies that included healthcare staff perspectives explored using translated PROMs when caring for people from CALD backgrounds.

#### Theme: Availability of culturally congruent and linguistically correct PROMs was important

Surgeons felt leadership advocacy and financial resources determined whether using translated PROMs was feasible in clinical care [34]. Surgeons reflected that access to culturally congruent and linguistically correct PROMs was important so that no matter the person’s culture or language, questions were interpreted in the same way and meaningful to the patient’s lived experience: *“something just transcribed from English to another language does not necessarily equate to what the intention of the questionnaire was.” (Surgeon)* [34].

#### Theme: PROMs should add value to existing clinical practice

Both studies that reported on the perspectives of healthcare staff highlighted that the effort to use translated PROMs should coincide with the added value to biomedical evaluations [34, 38]. One surgeon wanted to see the benefit of PROM use more clearly to encourage them to “buy in” to using translated PROMs: *“it’s getting patients to buy in, but it’s also getting us docs to buy in” (Surgeon)* [34].

#### Theme: ePROMs can promote the use of translated PROMs in existing workflows

As electronic medical records (EMR) were in English, the ability to embed translated PROMs into the EMR was important for some healthcare staff. For surgeons, this was because it would improve the accessibility of PROM data, encourage the use of translated PROMs, and reduce the need for interpreters [34]. Dermatologists in Europe reported ePROMs were highly acceptable as the system reduced resources required to obtain and administer translated PROMs in clinics and data from multiple surveys was safely stored in a central system [38].

Based on the results reported in the included papers, strategies to support and promote PROM completion by people from CALD backgrounds and Indigenous Peoples are reported in Table 5.

**Table 5:** Summary of recommendations to support and increase PROM completion, by people from CALD backgrounds and Indigenous Peoples, based on evidence from included studies

Stage	Recommendations
During PROM development for CALD and Indigenous populations	• Consider the needs of individual populations and whether cultural and linguistic adaptations are required, rather than just language translation [34]. • PROMs should be developed free from medical jargon, suitable for patients with low health literacy [37]. • PROMs should be co-designed with communities to ensure questions align with culture and lived experiences [37]. • Increase financial resources and leadership support to embed translated PROMs in clinical workflows [34, 38].
During electronic PROM system development	• Ensure electronic systems are accessibly designed prior to release (e.g., minimal system errors, user friendly for patients, embedded in electronic medical records for healthcare staff, result outputs for translated PROMs are provided in English for healthcare staff) [36, 38].
During patient communication about PROM completion (i.e., explaining purpose, explaining importance).	• Offer patients different modes of PROM completion based on personal preference. Consider developing PROMs that can be completed verbally [37]. • Healthcare staff should explain to family and carers the importance of reading PROM questions and responses *in verbatim* to patients [34–36]. • The purpose and importance of PROMs and the meaning of questions should be communicated to patients prior to completion (e.g., video resources, via healthcare staff, community members, etc.) [37].
After PROM completion	• Healthcare staff should help patients understand how PROM data relates to health-related quality of life, therapeutic options, or when compared to average scores for the relevant patient cohorts.

Abbreviations: CALD: culturally and linguistically diverse; PROMs: patient-reported outcome measures.

## Discussion

This study synthesises strategies of PROM completion by CALD and Indigenous Peoples and their healthcare teams. The main findings were (1) offering different modes of completion could facilitate PROM completion by accommodating varied health and digital literacy levels, (2) patients required assistance to understand and complete PROMs, and (3) surgeons believed culturally and linguistically appropriate translations of PROMs were important but difficult to obtain and embed in clinical workflows. These findings are consistent with other systematic reviews reporting the barriers and enablers for PROM implementation in English-speaking populations [39].

Some unique findings, specific to Indigenous Peoples were (1) the content of PROMIS-29 and PAID was not acceptable to Aboriginal and Torres Strait Islander Peoples who speak Australian Aboriginal English rather than Standard English, and (2) verbal completion may be a culturally appropriate mode of completion for Aboriginal and Torres Strait Islander Peoples. Further research is required to determine how these strategies could be implemented with other Indigenous populations, and whether they could be adopted for CALD populations.

The Agency of Clinical Innovation (Sydney, Australia), our project partner, collect ePROMs in 11 community languages in New South Wales (NSW) using a digital platform- the Health Outcomes and Patient Experience (HOPE). At the time of publication, over 108,000 PROMs have been collected using HOPE, which has been translated into ten community languages other than English. Given the success of this large-scale, digitally enabled collection of PROMs, the blueprint for this program has been adopted by other Australian states. With this in mind, we considered how enablers reported in this review can be feasibly scaled into large-scale PROM initiatives for CALD and Indigenous populations globally.

Offering PROMs in a range of community languages may overcome some reported challenges such as circumnavigating language and cultural barriers during clinical encounters (e.g., assessing pain), especially if an interpreter is unavailable. Reflecting the importance of this, HOPE was co-designed with patients and carers.

To be meaningful to patients and useful in clinical practice, evidence suggests PROMs should not only be translated but also culturally and linguistically adapted to ensure acceptability and appropriateness amongst people who are CALD, Indigenous Peoples and healthcare staff [38], and applicable in clinical care settings. Across cultural groups in this study, there was mixed evidence about the importance of culturally and linguistically adapted PROMs. For Aboriginal and Torres Strait Islander peoples in this study, PROMIS-29 did not align with their lived experience, values, and daily priorities [37]. On the contrary, one study set in an European dermatology clinic did not report evidence their ePROM system was culturally adapted or appropriate, or if there was training provided to patients to complete ePROMs [38]. Nevertheless, the study reported that people from CALD backgrounds found ePROMs easy to use [38]. Further, evidence is required to elucidate if translations are culturally appropriate or if cultural adaption is necessary to promote the acceptability of PROMs within a European context and other CALD populations. Our review reported that when patients had assistance to complete PROMs, questions and responses were not always read precisely [36]. This emphasises the importance of reading PROMs in verbatim to minimise interpretation errors, which can contribute to inaccurate responses and, therefore, misinformed clinical care. Importantly, for people who are CALD and Indigenous Peoples to self-complete PROMs, administrative instructions should also be translated into their preferred language, which facilitated the reported success of one included study in an European dermatology setting [38] and is also a feature of HOPE. Integration of translated PROM data with patients’ EMR would facilitate PROM use by healthcare staff and enable linkage of PROs to clinical events to facilitate timely care [40], a feature available in HOPE.

There may also be cultural barriers to PROM completion that can be overcome with novel developments in PROM administration, such as verbal completion in a patient’s preferred language. For example, ‘yarning’ builds a culturally safe environment for Aboriginal and Torres Strait Islander peoples by giving the community a space to talk, share, educate and build relationships [41, 42]. Yarning can facilitate culturally competent research and healthcare by emphasising two-way communication between health practitioners (or researchers) and participants [41, 42]. PROMs could be collected while Aboriginal and Torres Strait Islander peoples yarn, fostering trust between healthcare staff and patients [37].

### Implications on practice, policy, and future research

Findings from this review have implications for practice and policy as several recommendations to assist the completion of PROMs by CALD and Indigenous Peoples have been made. In future research, in partnership with NSW Health, we will explore the gap in documented knowledge about factors that determine the acceptability and suitability of PROMs to diverse cultural and linguistic populations [43]. By addressing these challenges, we can move towards a more equitable healthcare system that respects and responds to the unique needs of all patients, regardless of cultural or language background.

### Limitations

Notably, few studies met the eligibility criteria for this review. This was primarily because papers: (1) mentioned CALD and/or Indigenous terms in the title or abstract, but not within the study design; and/or (2) did not use PROMs as their intervention (3) reported only barriers and not strategies. The quality of the evidence using the MMAT appraisal tool was low, highlighting the need for high-quality, robust evidence of specific strategies of PROM completion in CALD and Indigenous populations. Incongruent definitions or terms to describe CALD and Indigenous populations were used in the literature. To mitigate this, we ensured our search strategy was comprehensive and incorporated terms used globally to describe these populations. The search was restricted to published scientific literature; it may be possible that relevant learnings from grey literature were missed.

## Conclusion

Some PROMs do not reflect the priorities and lived experiences of people who are CALD or Indigenous Peoples, leading to poor uptake. Consultation with people from a CALD background and Indigenous Peoples during PROM development and implementation could improve completion ensuring the content, mode of administration and implementation approaches were culturally appropriate and acceptable. Further, to successfully promote the completion of translated PROMs within chronic care settings, adequate training, planning, resourcing, and financial support are required for healthcare teams. Improving the completion rates of PROMs by people who are CALD and Indigenous Peoples will contribute to a more comprehensive and equitable assessment of patient needs, better informed clinical decisions, and a significant enhancement in these individuals’ overall quality of life.

## Data Availability

All included papers can be sourced in the reference list and data is available in the tables included in this manuscript.

## Abbreviations

CALD: Culturally and linguistically diverse
PRO: Patient-reported outcome
PROM: Patient-reported outcome measures
HRQL: Health-related quality of life
PRISMA: Preferred Reporting Items for Systematic Reviews and Meta-Analyses
CASP: Critical Appraisal Skills Programme
MMAT: Mixed Methods Appraisal Tool
NSW: New South Wales
PAID: Problem Areas in Diabetes
PROMIS-29: Patient-Reported Outcomes Measurement Information System-29
ePROMs: Electronic patient-reported outcome measures
EMR: Electronic medical records
